# Performance evaluation of the sysmex XN-1000V automated hematology analyzer in marine fish species: Gilthead seabream *(Sparus aurata)*

**DOI:** 10.1371/journal.pone.0350278

**Published:** 2026-07-06

**Authors:** Montse Mesalles, Josep Pastor, Emmanuel Serrano, Rafaela Cuenca, Lluís Tort, Mariana Teles

**Affiliations:** 1 Departament de Medicina i Cirurgia Animals, Facultat de Veterinària, Servei d’Hematologia Clínica Veterinària (SHCV), Universitat Autònoma de Barcelona (UAB), Cerdanyola del Vallès, Barcelona, Spain; 2 Departament de Medicina i Cirurgia Animals, Facultat de Veterinària, Wildlife Ecology & Health Group (WE&H), Servei d’Ecopatologia de Fauna Salvatge (SEFaS), Universitat Autònoma de Barcelona (UAB), Cerdanyola del Vallès, Barcelona, Spain; 3 Departament de Biologia Cel·lular, Fisiologia i Immunologia, Facultat de Biociències, Universitat Autònoma de Barcelona (UAB), Cerdanyola del Vallès, Barcelona, Spain; University of Kashmir, INDIA

## Abstract

Hematological analyses are widely used to assess the health and physiological status of animals, including fish. Automated hematology analyzers enable rapid and reproducible hematological measurements; however, their application in fish requires species-specific analytical validation due to the presence of nucleated blood cells. This study evaluated the analytical performance of the Sysmex XN-1000V hematology analyzer operated under avian settings (PLT-F channel), in juvenile gilthead seabream (*Sparus aurata*), one of the cornerstone species of Mediterranean marine aquaculture. Analytical validation included assessment of precision, linearity, carryover, and sample stability. Results were compared with manual reference methods (hemocytometer counts and blood smear-based estimates). In addition, the influence of two anticoagulants (lithium heparin and K₂EDTA) was examined, and the analyzer’s ability to detect lipopolysaccharide (LPS)-induced changes in non-erythroid cell population was evaluated. The analyzer showed good precision for most parameters (CV < 5%), although higher variability was observed for granulocyte percentage. Linearity was excellent (R^2^ ≥ 0.98), and carryover remained below 1% for all variables. Sample stability was significantly better at 4 °C than at room temperature (25 °C), supporting refrigerated storage for up to 24 hours prior to analysis. Comparison with manual reference methods revealed strong correlations and agreement for red blood cell counts and hematocrit, whereas discrepancies were identified in non-erythroid cells differentials. Among anticoagulants, lithium heparin yielded more consistent and reliable results than K₂EDTA. Erythrocyte-related parameters showed the highest analytical reliability, whereas the leucocyte differentiation within the non-erythroid fraction required complementary microscopic evaluation, particularly when assessing LPS-induced inflammatory changes. Overall, the Sysmex XN-1000V demonstrated suitable analytical performance for routine hematological assessment in gilthead seabream when operated under avian settings. These findings support its application in aquaculture health monitoring programs, provided that appropriate anticoagulants are used, samples are promptly processed or refrigerated, and automated results are complemented by microscopic review when leukocyte counts are suspected.

## Introduction

Hematological analyses are recognized as valuable tools for assessing the physiological condition and overall health status of vertebrates, including fish, in both wild and cultured populations [1–4]. Unlike mammals, fish possess fully nucleated erythrocytes and thrombocytes, a feature shared with avian hematology, which complicates automated discrimination and classification [5]. As a result, manual hematological methods are still commonly used, although they are inherently operator-dependent.

Automated hematology analyzers have become important diagnostic tools in veterinary medicine [6], including applications in aquatic species. These instruments provide rapid, standardized, and reproducible evaluations of blood cells, reducing operator-dependent variability and enhancing diagnostic accuracy. However, the application of automated hematology systems in fish presents specific challenges.

The Sysmex XN-1000V is an automated hematology analyzer equipped with multi-species software specifically designed for veterinary applications and validated in a wide range of mammalian species [7–9] and, more recently, in selected non-mammalian species [10,11]. The system combines electrical impedance, laser light scattering, and fluorescence flow cytometry to differentiate erythrocytes, leukocytes, and platelets. Fish blood cells are nucleated, and analytical settings developed for avian hematology are commonly used as a reference profile when analyzing fish blood samples.

Automated hematology has been explored in several teleost species, demonstrating promising analytical performance [12–14], but its applicability to marine aquaculture species remains uncertain. Mesalles et al. [15] have recently validated the Sysmex XN-1000V for the freshwater salmonid species *Oncorhynchus mykiss*. Physiological differences between marine and freshwater teleosts, including plasma osmolarity and erythrocyte characteristics, may influence the analytical performance of automated hematology analyzers.

Evidence indicates that the optimal anticoagulant for fish blood samples often depends on species-specific physiological factors [16–18]. It is essential to consider the potential effects of EDTA and heparin on hematological results obtained with the Sysmex XN-1000V to ensure accurate interpretation of data and guide the selection of the most appropriate anticoagulant for *Sparus aurata*, depending on the parameter of interest [19,20].

In addition to analytical validation, the analyzer’s ability to detect shifts in the distribution of non-erythroid cell populations during inflammatory responses is also relevant for fish health monitoring in aquaculture systems. Intraperitoneal administration of bacterial lipopolysaccharide (LPS) is widely used in teleost fish as a robust model for inducing innate immune activation, eliciting inflammatory leukocyte recruitment, and altering circulating leukocyte profiles [21,22].

To fill this gap of knowledge, this study evaluates the analytical performance of the Sysmex XN-1000V hematology analyzer in gilthead seabream (*Sparus aurata*), a euryhaline marine teleost of major economic importance in European aquaculture, valued for its rapid growth and adaptability [23]. In particular, we will assess precision, linearity, carryover, and sample stability using the PLT-F channel and avian settings and compare automated measurements with manual reference methods in accordance with international guidelines [24,25]. In addition, the effects of two commonly used anticoagulants, K₂EDTA and lithium heparin, were examined to identify the most suitable anticoagulant for hematological analyses in this species. Finally, the study investigated the analyzer’s ability to detect shifts in non-erythroid cell populations during LPS-induced inflammation.

## Materials and methods

### Animals

One hundred and twelve juvenile gilthead seabreams (weight: 13.69 ± 5.73 g; length: 10.35 ± 1.53 cm) were obtained from the fish farm Avramar España Acuicultura S. L. (Alicante, Spain). Fish were transported to the facilities of the Universitat Autònoma de Barcelona (AQUAB-Fish) and acclimated for 20 days in a closed recirculating seawater system with a 1000 L tank, thermoregulated at 18–20 °C under a 12:12h light: dark photoperiod. Water quality parameters (temperature, dissolved oxygen, pH, nitrite, and ammonia) were regularly monitored and maintained within species-appropriate ranges. Fish were fed daily with a commercial pellet diet (INTRO Plus MT, BioMar Iberia, Spain) at 2% of body weight. All fish were clinically healthy, based on the absence of observable clinical signs of disease and normal physiological condition at the time of sampling. Specifically, they exhibited (i) normal external appearance (no skin lesions, ulcers, hemorrhages, or fin erosion), (ii) normal behavior (active swimming and feeding response), and (iii) absence of visible parasites upon routine inspection. No parasitological or bacteriological tests were performed.

All procedures followed the International Guiding Principles for Biomedical Research Involving Animals (EU Directive 2010/63) and were approved by the Universitat Autònoma de Barcelona Ethics Committee (permits OH4218 and DAMM11251).

### Blood collection

Randomly selected fish were anesthetized with buffered tricaine methane sulfonate (MS-222; Sigma-Aldrich, Merck KGaA, Darmstadt, Germany) at a lethal dose of 0.8 g/L with 1.6 g/L NaCl. Blood was collected by puncturing the caudal vein using 25G × 1.5-inch needles and heparin-coated insulin syringes and then transferred to 1.5 ml microtubes containing lithium heparin (Li-heparin; Sigma-Aldrich, Merck KGaA). Samples were stored at 4 °C, analyzed by the same operator within two hours of collection, except those reserved for stability testing, and processed using both automated and manual methods. Before analysis, refrigerated samples were brought to room temperature and gently mixed on a rotator (VSR23, Grant Instruments Ltd., Cambridge, UK) for 10 minutes to ensure full homogenization.

### Automated and manual hematological methods

#### Sysmex XN-1000V, hematology analyzer.

**Fig 1:**
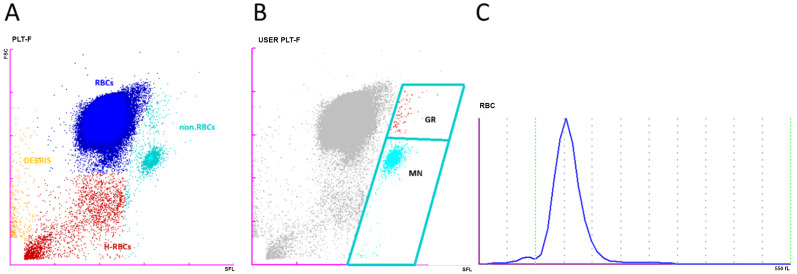
Cell population distributions and erythrocyte volume in gilthead seabream. (A) Scatter plot from the PLT-F channel of the Sysmex XN-1000V analyzer showing the distribution of blood cell populations in gilthead seabream: red blood cells (RBCs, blue), non-erythrocyte cells (non-RBCs, turquoise), hemolyzed red blood cells (H-RBCs, red), and debris (orange). (B) Scatter plot illustrating the manual gating strategy used to distinguish mononuclear (MN) and granulocyte (GR) cell populations. (C) Histogram showing the distribution of erythrocyte volume parameters, including mean corpuscular volume (MCV).

Whole blood samples were analyzed using a Sysmex XN-1000V hematology analyzer (Sysmex Co., Kobe, Japan) equipped with multispecies software (version 3.05). This instrument integrates impedance and optical fluorescence technologies. Instrument settings, gating strategies, and sample handling procedures followed those previously described by our group in rainbow trout [15]. Representative scatterplots from the PLT-F channel for gilthead seabream are shown in Fig 1, illustrating the classification of the main cell populations: red blood cells (RBCs) and non-erythroid cells (non-RBCs).

Internal quality control (QC) was performed daily using Sysmex XN Check Level 2 QC material (Sysmex Co., Kobe, Japan). The QC sample was run at the start of each working day, and results were accepted according to the manufacturer’s specifications. All samples were analyzed by the same operator. All blood samples were analyzed individually for every validation experiment, and each fish was treated as an independent biological replicate; no pooling was performed at any stage of the study.

#### Manual counts and smear examination of gilthead seabream blood samples.

Manual red blood cell (RBC) and non-RBC counts were performed following our standardized clinical laboratory protocol [15]. Briefly, blood samples were diluted 1:200 in commercial Natt and Herrick diluent (Bioanalytic GmbH, Germany), incubated briefly, and counted in duplicate using an improved Neubauer hemocytometer. Packed cell volume (PCV) was determined using the microhematocrit technique, in which microcapillaries were filled to three-quarters of their length with blood and centrifuged at 13,143 × g for 5 minutes (Centromix II BL, J.P. Selecta S.A., Spain). After centrifugation, capillaries were read using a microhematocrit reader (Hawksley and Sons, S.L., England), and results were expressed as percentages. A subjective estimation of the number of non-red blood cells (non-RBCs) per mm³ was obtained from a peripheral blood smear by calculating the mean number of non-RBCs in ten high-power fields (60×) and multiplying the result by 2,000. Differential counts of non-erythroid cells were performed on peripheral blood smears stained with modified Romanowsky (Panoptic, QCA S.A., Tarragona, Spain). A total of 100 non-erythrocytic cells were examined at 1000 × magnification using a Nikon Eclipse Ci microscope, and classified as mononuclear (lymphocytes, monocytes, thrombocytes) or granulocytic cells (heterophils, eosinophils).

#### Validation of the Sysmex XN-1000V for gilthead seabream blood samples.

For the validation of the Sysmex XN-1000V automated hematology analyzer in gilthead seabream, the following parameters were assessed: RBC count, hemoglobin concentration, hematocrit/packed cell volume (PCV), mean corpuscular volume (MCV), non-RBC count, and the relative proportions of granulocytes and mononuclear cells.

Within-run precision, linearity, carryover, sample stability, and method comparison were evaluated on the Sysmex XN-1000V using blood samples from gilthead seabream.

For precision evaluation, three heparinized blood samples representing high, normal, and low hematologic values were analyzed in triplicate. The precision of the manual method for RBC and non-RBC counts was also assessed in triplicate. Counting was performed from the initial dilution step through microscopic examination using an improved Neubauer hemocytometer, and all analyses were carried out by a single trained operator.

Linearity was assessed by measuring serial dilutions (100%, 75%, 50%, and 25%) of a concentrated heparinized blood sample in duplicate. The sample was centrifuged at 1,600 × g for 10 minutes, after which 80% of the plasma was removed to prepare the dilutions. Cellpack-DCL diluent (Sysmex Co., Kobe, Japan) was used as the diluent. To confirm the absence of analytical interference, the instrument diluent was analyzed in triplicate.

Carryover was evaluated by analyzing a concentrated blood sample in triplicate, followed by a low-level sample prepared with Cellpack-DCL. For the stability assessment, blood samples from 22 gilthead seabreams were collected into lithium-heparin tubes and immediately analyzed (T0). Nine samples were stored at 4 °C and 13 samples at room temperature (approximately 25 °C). Each sample was reanalyzed by the same operator after 24 and 48 hours of storage. Before analysis, refrigerated samples were brought to room temperature and gently mixed for 10 minutes to ensure homogeneity.

For the method comparison, fifty-seven blood samples collected from juvenile gilthead seabream were analyzed to compare automated and manual methods, including RBC counts, hematocrit (HCT)/packed cell volume (PCV), non-RBC counts obtained by automated analysis, manual hemocytometer counts, and smear-based estimate, and the percentages of granulocytes and mononuclear cells determined by both automated analysis and manual smear evaluation.

#### Impact of anticoagulant.

Twenty blood samples were collected from gilthead seabream into two types of tubes: lithium-heparin (n = 10; Sigma-Aldrich, Merck KGaA) and dipotassium EDTA (n = 10; K₂EDTA, BD Vacutainer, Becton Dickinson).

All samples were analyzed using the Sysmex XN-1000V immediately after collection by a single operator under identical instrument settings.

Hematological parameters were then compared to evaluate the impact of anticoagulant type.

#### Evaluation of non-erythroid cell distribution following experimental LPS stimulation.

Four gilthead seabream were intraperitoneally injected with *Escherichia coli* LPS (7 mg/kg; Sigma, Alcobendas, Spain), while six control fish received saline injections. Twenty-four hours post-injection, blood samples were collected from the caudal vein, placed into heparinized syringes, and immediately analyzed using the Sysmex XN-1000V.

To determine the differential leukocyte count (granulocytes and mononuclear cells), blood smears were prepared using a standard two-slide wedge technique, air-dried, and stained with a modified Romanowsky stain (Panoptic, QCA S.A., Tarragona, Spain). Smears were then examined by light microscopy (Nikon Eclipse Ci, Nikon Instruments, Tokyo, Japan).

### Statistical analysis

Normality was assessed using the Shapiro–Wilk test. Parameters that conform to a normal distribution were analyzed using parametric methods, whereas non-normally distributed parameters were analyzed using nonparametric tests. Precision was expressed as the coefficient of variation (CV), mean, and standard deviation. Linearity was evaluated by linear regression (Pearson’s r). Storage effects were assessed using the Friedman test followed by Dunn’s multiple comparisons test. Agreement between the Sysmex XN-1000V and manual reference methods was evaluated using Pearson correlation, Passing–Bablok regression, and Bland–Altman analysis (via https://bahar.shinyapps.io/method_compare/). The effect of anticoagulant type (Li-heparin vs K₂EDTA) was assessed using the paired Wilcoxon signed-rank test. Analyzer sensitivity to LPS-induced inflammation was evaluated using the Mann–Whitney U test. All statistical analyses were performed in GraphPad Prism v8.0.1 (GraphPad Software, San Diego, CA, USA). Statistical significance was set at p < 0.05.

## Results

### Repeatability (Within-run precision)

Within-run precision results are presented in Table 1. Most parameters showed acceptable repeatability across both low-to-normal and high cell count ranges. Granulocyte percentage showed the greatest variability at low-to-normal concentrations, with a coefficient of variation (CV) exceeding 5%.

**Table 1:** Within-run precision of hematological parameters measured using the Sysmex XN-1000V analyzer in *Sparus aurata.*

Parameter (unit)	Low mean	CV (%)	Normal mean	CV (%)	High mean	CV (%)
RBC (×10⁶/µL)	0.48	4.19	1.89	1.32	2.05	0.49
Hb (g/dL)	0.93	4.28	2.96	1.71	4.23	1.42
Hct (%)	7.00	1.16	25.30	0.40	35.06	0.33
MCV (fL)	107.20	0.37	131.30	2.11	152.90	0.27
non-RBC (×10³/µL)	35.33	1.53	56.41	1.29	109.49	3.74
Mononuclear cells (%)	26.80	3.99	60.04	3.72	97.57	0.36
Granulocytes (%)	3.23	9.30	39.96	5.59	73.00	3.33

**Abbreviations:** RBC, red blood cells; Hb, hemoglobin; Hct, hematocrit; MCV, mean corpuscular volume; CV, coefficient of variation; non-RBC, non-erythrocyte cells.

Manual precision for red blood cell (RBC) counts was high when performed by the same operator (CV = 1.03%). In contrast, non-RBC counts showed greater variability (CV = 5.39%) (Table 2).

**Table 2:** Manual RBC and non-RBC counts determined by the same operator in a single *Sparus aurata* blood sample.

Parameter (unit)	Mean	CV (%)
RBC (×10⁶/µL)	2.44	1.03
non-RBC (×10³/µL)	45.53	5.39

**Abbreviations:** RBC, red blood cells; non-RBC, non-erythrocyte cells; CV, coefficient of variation.

### Linearity

**Fig 2:**
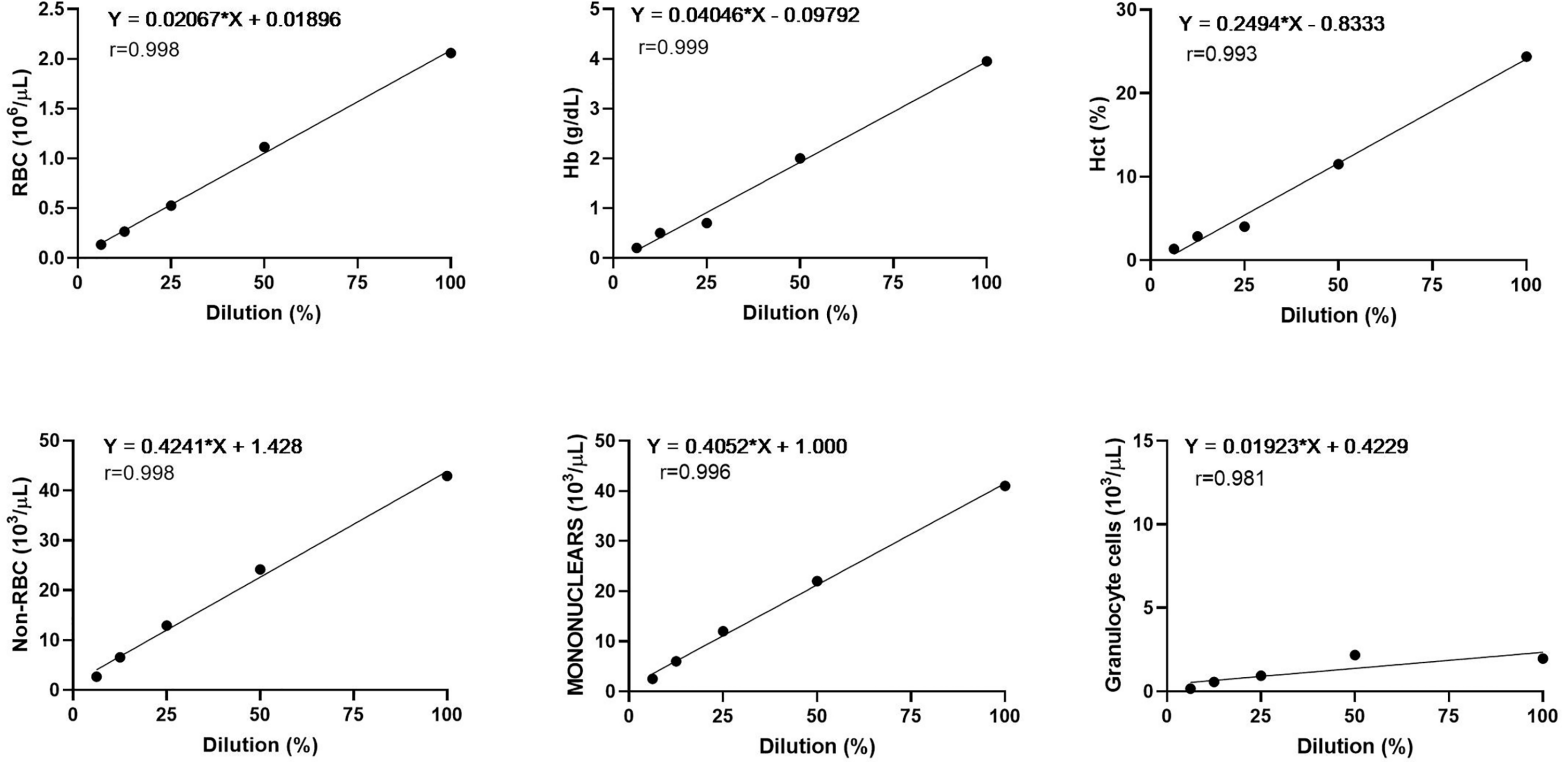
Linearity analysis of hematological parameters in gilthead seabream. Linearity was evaluated for red blood cell (RBC) count, hemoglobin concentration (Hb), hematocrit (Hct), non-erythrocyte cell count (non-RBC), mononuclear cells, and granulocytes measured from a single blood sample of gilthead seabream. Each plot shows the regression line and the Pearson correlation coefficient (R^2^).

Linearity of the Sysmex XN-1000V was excellent for all evaluated parameters, with coefficients of determination (R^2^) greater than 0.99, except for granulocyte counts (Fig 2).

### Carryover

Carryover values were within the manufacturer’s acceptable limits (< 1%).

### Stability assessment

**Fig 3:**
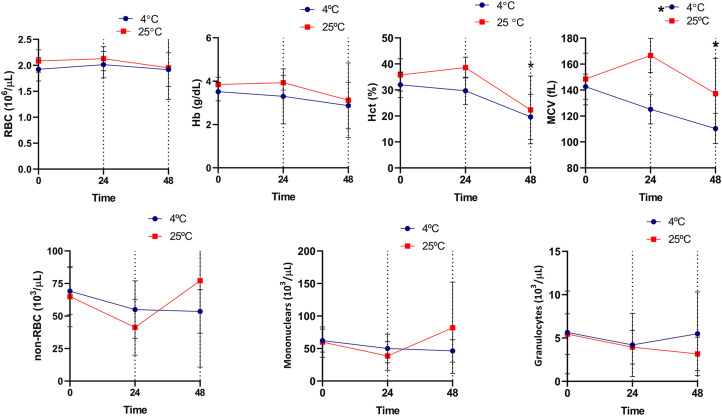
Stability of hematological parameters in gilthead seabream blood samples stored at 4 °C and 25 °C. Hematological parameters were evaluated in blood samples stored at 4 °C (blue circles; n = 9) and 25 °C (red squares; n = 13) using the Sysmex XN-1000V analyzer. Plots show the mean ± SD for red blood cell (RBC) count, hemoglobin (Hb), hematocrit (Hct), mean corpuscular volume (MCV), non-erythrocyte cell (non-RBC) count, mononuclear cells, and granulocytes at 0, 24, and 48 h. Asterisks indicate statistically significant differences from baseline (T0) (Friedman test followed by Dunn’s multiple comparisons test, p < 0.05).

**Fig 4:**
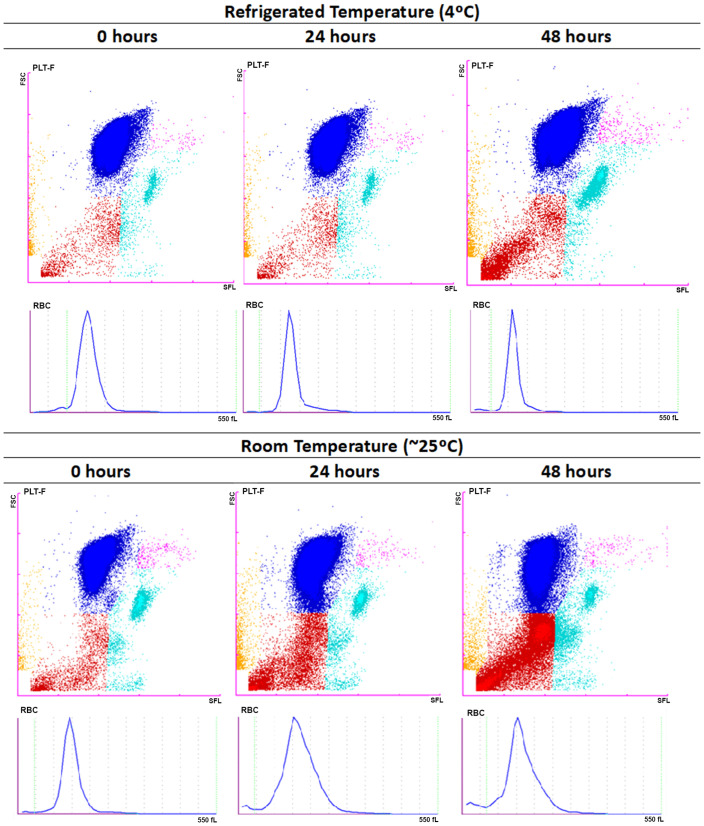
Representative PLT-F scattergrams and RBC histograms from two gilthead seabream samples stored at 4 °C or 25 °C. Scattergrams and histograms are shown at 0, 24, and 48 h, with red blood cells (RBCs, blue), granulocytes (pink), mononuclear cells (turquoise), hemolyzed red blood cells (H-RBCs, red), and debris (orange).

Figs 3 and 4 illustrate the effect of storage temperature (4 °C vs 25 °C) on blood samples. Statistical comparisons were performed against baseline values (T0). Erythrocyte counts and hemoglobin concentration remained stable over 48 hours at both temperatures. Hematocrit (Hct) values were stable during the first 24 hours but declined thereafter, with significant differences observed at 48 hours (p < 0.05). Mean corpuscular volume (MCV) increased in samples stored at 25 °C and decreased in those stored at 4 °C, with significant differences detected at both 24 and 48 hours (p < 0.05). Non-RBC and mononuclear cell counts showed transient fluctuations over time (p < 0.05), decreasing during the first 24 hours and increasing thereafter, whereas granulocyte counts showed a slight decline in samples stored at 25 °C. PLT-F scattergrams from samples stored at 25 °C showed broadened cell clusters and increased hemolyzed cells and debris, consistent with the observed numerical changes. In contrast, refrigerated samples exhibited well-preserved and compact distributions. Cytogram analysis indicated erythrocyte swelling and hemolysis at room temperature, associated with late stage increases in non-RBC counts, including elevated debris and cell fragments.

### Method comparisons

**Fig 5:**
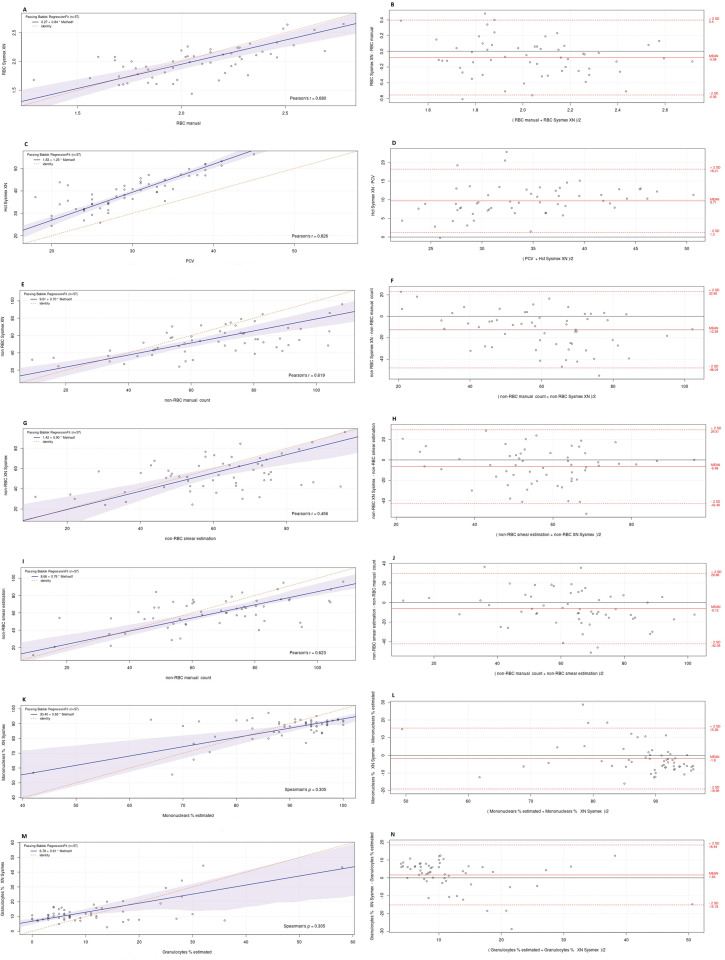
Comparison of hematological parameters measured by the Sysmex XN-1000V and manual reference methods in juvenile gilthead seabream. Passing–Bablok regression and Bland–Altman analyses were performed using blood samples from juvenile gilthead seabream (Sparus aurata, n = 57). Panels (A, C, E, G, I, K, M) show Passing–Bablok regression analyses with regression lines and 95% confidence intervals; slope and intercept estimates with their corresponding 95% confidence intervals are indicated in each panel. Panels (B, D, F, H, J, L, N) show Bland–Altman plots with the mean difference (bias, solid line) and the 95% limits of agreement (dashed lines). Evaluated parameters include red blood cell count (RBC, × 10⁶ cells/μL), packed cell volume (PCV, %), non-erythrocyte cell count (non-RBC, × 10³ cells/μL), and mononuclear and granulocyte populations (%).

The results of the method comparison are presented in Table 3 and Fig 5. The level of correlation and agreement between the Sysmex XN-1000V and manual results varied by parameter. RBC counts showed the strongest agreement, with a Pearson correlation of *r* = 0.68 and minimal mean bias, indicating only minor proportional discrepancies. Hematocrit/packed cell volume (Hct/PCV) also correlated well (r = 0.83), although values were overestimated at higher levels, indicating significant proportional bias. Non-RBC counts showed moderate correlation but weak agreement, with notable variability and bias, particularly when comparing analyzer-derived values with smear-based estimates, suggesting substantial differences between methods. Differential percentages of mononuclear cells and granulocytes provided by the analyzer showed only moderate agreement with smear estimates and exhibited consistent proportional errors with relatively wide limits of agreement.

**Table 3:** Method comparison between the Sysmex XN-1000V analyzer and manual reference methods for hematological parameters in gilthead seabream (*Sparus aurata,* n = 57).

Parameter (unit)	Method comparison	Pearson’s r	Passing–Bablok slope (95% CI)	Passing–Bablok intercept (95% CI)	Bland–Altman mean bias	Lower LoA	Upper LoA
RBC (×10⁶/µL)	Sysmex XN-1000V vs Manual count	0.68	0.84 (0.66–1.08)	0.27 (−0.23–0.67)	−0.08	−0.56	0.40
Hct/ PCV (%)	Sysmex XN-1000V vs PCV (microhematocrit)	0.83	1.25 (1.09–1.45)	1.83 (−4.35–6.67)	9.71	1.20	18.21
non-RBC (×10³/µL)	Sysmex XN-1000V vs Manual count	0.62	0.69 (0.50–0.95)	9.61 (−5.09–19.94)	−12.66	−48.04	22.85
non-RBC (×10³/µL)	Manual count vs Smear estimation	0.62	0.76 (0.58–0.92)	8.66 (−3.67–23.61)	−6.12	−42.09	29.86
non-RBC (×10³/µL)	Sysmex XN-1000V vs Smear estimation	0.46	0.90 (0.57–1.26)	1.42 (−24.14–21.73)	−6.48	−42.46	29.51

**Abbreviations**: RBC, red blood cells; Hct, hematocrit; PCV, packed cell volume; non-RBC, non-erythrocyte cells; LoA, limits of agreement; CI, confidence interval.

### Impact of anticoagulant type

**Fig 6:**
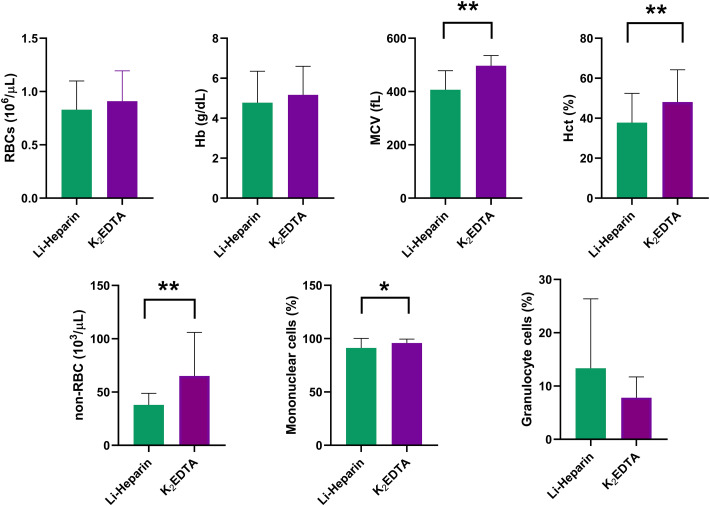
Impact of anticoagulant type on hematological parameters in juvenile gilthead seabream. Bar plots show the mean ± SD for paired blood samples collected in lithium heparin (Li-heparin; green) and dipotassium EDTA (K₂EDTA; purple) tubes (n = 10). Parameters evaluated include red blood cell (RBC) count, hemoglobin (Hb), hematocrit (Hct), mean corpuscular volume (MCV), non-erythrocyte cell (non-RBC) count, and mononuclear and granulocyte populations (%). Statistical comparisons were performed using the paired Wilcoxon signed-rank test, p < 0.05). Asterisks indicate significance: * p < 0.05; ** p < 0.01.

**Fig 7:**
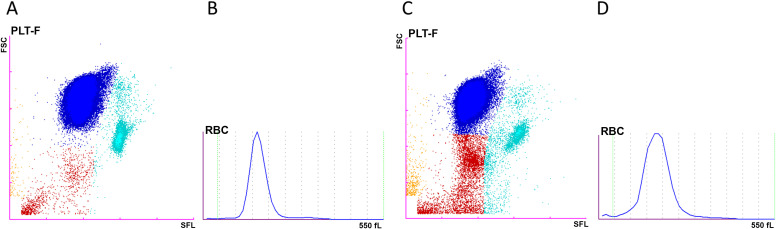
Scatter plots and histograms of blood samples collected with Li-heparin and K₂EDTA. Panels show PLT-F scatter plots (A, C) and mean corpuscular volume (MCV) distribution histograms (B, D) from paired blood samples collected in lithium heparin (A, B) and dipotassium EDTA (C, D). Cell populations are displayed as red blood cells (RBCs, blue), non-erythrocyte cells (non-RBCs, turquoise), hemolyzed red blood cells (H-RBCs, red), and debris (orange).

The effect of anticoagulant type on hematological parameters is shown in Fig 6. Hematocrit (Hct), mean corpuscular volume (MCV), total non-RBC count, and the percentage of mononuclear cells were significantly higher in K₂EDTA samples (Wilcoxon signed-rank test, p < 0.05). In contrast, RBC counts, hemoglobin concentration, and granulocyte percentages were not significantly affected by anticoagulant type (p > 0.05). Representative PLT-F scattergrams and RBC volume histograms illustrating these differences are shown in Fig 7.

### Hematological response to LPS stimulation

**Fig 8:**
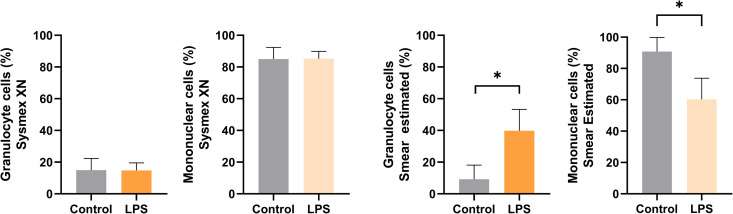
Hematological response to LPS stimulation in gilthead seabream. Bar plots show the mean ± SD for control (grey, n = 6) and LPS-treated (orange, n = 4) groups. Panels illustrate (A) granulocyte percentage measured by the Sysmex XN-1000V, (B) mononuclear cell percentage measured by the Sysmex XN-1000V, (C) granulocyte percentage from blood smear evaluation, and (D) mononuclear cell percentage from manual smear evaluation. Asterisks indicate significant differences between control and LPS-treated groups (Mann-Whitney U test, p < 0.05)..

Stimulation with LPS (7 mg/kg for 24 hours) did not produce significant changes in the hematological parameters assessed by the Sysmex XN-1000V between control and LPS-treated groups, including the relative numbers of mononuclear cells and granulocytes. In contrast, manual smear evaluation revealed significant changes in cell population proportions: the percentage of mononuclear cells decreased, whereas the percentage of granulocytes increased in the LPS-treated group (Fig 8).

## Discussion

In this work, we evaluated the performance ot the Sysmex XN-1000V hematology analyzer in gilthead seabream, a marine teleost species of major relevance for aquaculture. The analyzer enables rapid, reliable, and reproducible measurement of standard blood parameters and has recently been validated in fish. Mesalles et al. [15] provided the first evaluation of this analyzer in rainbow trout (*Oncorhynchus mykiss)*, demonstrating robust analytical performance.

However, the applicability of these findings across teleost species remains uncertain. Marine teleosts differ physiologically from freshwater species, and factors such as osmolarity, erythrocyte volume regulation, and leukocyte distribution patterns require species-specific validation rather than direct extrapolation [26].

Accordingly, this study addresses this gap by evaluating whether an analyzer validated in freshwater fish can be reliably applied to a marine teleost species.

The analyzer demonstrated analytical precision within internationally accepted performance criteria for both erythroid and non-erythroid parameters [24,25], confirming its technical repeatability under controlled conditions. The granulocyte-related parameter showed comparatively higher variability, consistent with previous observations in rainbow trout [15]. In teleost fish, the relatively low abundance and morphological diversity of granulocytes [27] may reduce the sensitivity of cluster discrimination and gating algorithms in fluorescence-based systems originally optimized for mammalian cells.

The strong linearity observed across the measurement range indicates a proportional signal response over clinically relevant concentrations, supporting the instrument’s quantitative reliability for use in this species. Furthermore, the minimal carryover (<1%) minimizes the risk of inter-sample contamination, which is particularly relevant in fish hematology due to the presence of nucleated cells.

Sample stability is a critical pre-analytical determinant of hematological reliability in teleost fish, as storage conditions directly influence membrane integrity and cellular morphology [18,28,29]. In our study, temperature and storage time significantly affected most hematological parameters, except for RBC count and hemoglobin concentration, which remained stable. Hematocrit decreased after 24 h at both temperatures, contrasting with the findings of Fazio et al. [12], likely reflecting methodological or instrument-related differences.

At 4 °C, erythrocyte shrinkage and reduced MCV were consistent with cold-induced membrane alterations**.** At 25 °C, osmotic imbalance and increased membrane permeability likely promoted erythrocyte swelling and lysis [28], explaining the increase in MCV. This pattern was also evident in the RBC histograms (Fig 4).

Non-erythroid fractions were particularly unstable at 25 °C, consistent with previous reports [12,15]. Progressive cellular degeneration and hemolysis likely generated debris and free erythrocyte nuclei, which may have been misclassified within the non-RBC cluster, thereby compromising leukocyte differentiation.

Refrigeration (4 °C) better preserved cellular integrity and limited pre-analytical artifacts, supporting prompt processing or short-term storage (<24 h) of blood samples in fish hematology [30–32].

Comparative analysis indicates that automated RBC counts show acceptable agreement with manual counts under the evaluated conditions, supporting their quantitative reliability in this species. In contrast, hematocrit values demonstrated systematic overestimation, consistent with osmotic differences between marine plasma (320–380 mOsm·kg ⁻ ¹ [33]) and the analyzer diluent (~250 mOsm·kg ⁻ ¹ [34]), which may promote erythrocyte swelling and increase mean corpuscular volume, leading to artefactual hematocrit elevation. Hematocrit should therefore be interpreted with caution and confirmed by microhematocrit when clinically relevant.

Discrepancies were most evident in the non-erythroid cell fraction. The analyzer systematically reported lower non-RBC counts than manual hemocytometer and smear-based estimates, indicating limited interchangeability for leukocytes and thrombocytes quantification in *Sparus aurata*.

This divergence likely reflects fish-specific pre-analytical and biological factors, including rapid clotting and cellular activation. Even under heparin anticoagulation, leukocyte–thrombocyte aggregation may occur. Such aggregation may be interpreted as single events or excluded by fluorescence-based detection, leading to underestimation. In contrast, manual methods allow visual recognition of clustered or morphologically altered cells.

In addition, morphological overlaps between immature thrombocytes and small mature lymphocytes [27], together with the low and highly variable proportion of granulocytes in fish peripheral blood [35,36], complicates algorithm-based discrimination. The low abundance of granulocytes, together with their morphological heterogeneity, may further limit the algorithm’s ability to identify a clearly defined cluster. Because automated systems are originally optimized for mammalian blood, when cluster separation is not clearly defined, manual gating adjustments may improve visual separation but can introduce operator-dependent variability.

Overall, these findings suggest that total non-RBC counts may serve as a general indicator of marked hematological alterations, whereas leukocyte subclassification should be interpreted cautiously and confirmed by smear evaluation, particularly under inflammatory conditions such as those assessed in our exploratory LPS model.

Pre-analytical factors, particularly anticoagulant selection, are essential to preserve sample integrity and ensure reliable analytical results [37]. This is especially important in fish, whose blood exhibits a pronounced clotting tendency [38]. Although no consensus exists regarding the optimal anticoagulant for fish hematology, there is broad agreement that suitability is species dependent. Additional validation studies assessing different anticoagulants in target species are warranted, as available data for marine teleosts, including *Sparus aurata,* remain limited compared with freshwater species.

When EDTA is used as an anticoagulant in this species, spuriously elevated hematocrit (Hct) and mean corpuscular volume (MCV) values are to be expected as previously described in fish [39–41]. This artifact arises from the mechanism of action of EDTA, which alters membrane stability and ion permeability, promoting Na⁺ influx and osmotic water entry [42]. Additionally, non-erythrocyte counts and the proportion of mononuclear cells are also likely to be overestimated. This effect can be attributed to the mildly hemolytic action of EDTA on erythrocytes in this species reflecting their inherent fragility and increased susceptibility to hemolysis [43,44], which may interfere with automated cell classification. This interpretation is supported by the scatterplots and histograms obtained in the present study (Fig 6 and 7). Similar analytical interference has previously been reported in *Oncorhynchus mykiss* [15].

Under the conditions evaluated, lithium heparin appeared to better preserve cellular morphology and analytical stability, but not artifact-free; anticoagulant suitability should be interpreted within a species-specific and methodological context.

Finally, regarding the study to investigate the analyzer’s ability to detect shifts in non-erythroid cell populations during LPS-induced inflammation, it must be taken into consideration that this experiment was exploratory, limited to a single time point and a small number of individuals. Given that teleost responses to endotoxin are known to vary with dose and timing [22,45], our results should be interpreted cautiously, and further studies are required to more fully characterize the analyzer’s performance under inflammatory conditions.

## Conclusion

The Sysmex XN-1000V analyzer may serve as a complementary tool for routine hematological monitoring in *Sparus aurata*, provided that smear verification and appropriate sample handling are implemented to ensure accurate interpretation. The analyzer demonstrated good precision and acceptable agreement between automated and manual RBC counts; however, hematocrit values should be interpreted with caution and verified by microhematocrit when clinically relevant. Manual examination of the blood smear remains necessary due to the analyzer’s limitations in leukocyte differentiation and its sensitivity for detecting inflammatory responses. Prompt analysis or short-term refrigerated storage of the blood samples is recommended when EDTA is used as the anticoagulant, whereas lithium heparin yields more consistent measurements under the conditions evaluated.

## References

[1] Maceda-Veiga A, Figuerola J, Martínez-Silvestre A, Viscor G, Ferrari N, Pacheco M. Inside the redbox: Applications of haematology in wildlife monitoring and ecosystem health assessment. Sci Total Environ. 2015;514:322–32. doi: 10.1016/j.scitotenv.2015.02.004 25668285

[2] Docan A, Grecu I, Dediu L. Use of hematological parameters as assessment tools in fish health status. J Agroaliment Process Technol. 2018;24:317–24.

[3] Seibel H, Baßmann B, Rebl A. Blood will tell: What hematological analyses can reveal about fish welfare. Front Vet Sci. 2021;8:616955. doi: 10.3389/fvets.2021.616955 33860003 PMC8042153

[4] Chen H, Luo D. Application of haematology parameters for health management in fish farms. Reviews in Aquaculture. 2022;15(2):704–37. doi: 10.1111/raq.12753

[5] Campbell TW, Grant KR. Exotic Animal Hematology and Cytology. 5th ed. Hoboken, NJ: Wiley-Blackwell. 2022.

[6] DeNicola DB. Advances in hematology analyzers. Top Companion Anim Med. 2011;26(2):52–61. doi: 10.1053/j.tcam.2011.02.001 21596345

[7] Grebert M, Granat F, Braun J-P, Leroy Q, Bourgès-Abella N, Trumel C. Validation of the Sysmex XN-V hematology analyzer for canine specimens. Vet Clin Pathol. 2021;50(2):184–97. doi: 10.1111/vcp.12936 34152026 PMC8362000

[8] Guerlin M, Granat F, Grebert M, Braun J-P, Geffré A, Bourgès-Abella N, et al. Validation of the Sysmex XN-V hematology analyzer for feline specimens. Vet Clin Pathol. 2024;53(3):294–308. doi: 10.1111/vcp.13377 39294107

[9] Schroeder JM, Lee H-YC, Schultze AE. Performance evaluation of the Sysmex XN-1000V in side-by-side comparison with the Siemens ADVIA 120 and manual methods for healthy CD Sprague-Dawley rats and CD-1 mice. Vet Clin Pathol. 2024;53(1):8–39. doi: 10.1111/vcp.13314 38164989

[10] Meazzi S, Martini V, Moretti A, Lubian E, Paltrinieri S, Giordano A. Automated hematological cell count using sysmex XN-1000V in Testudo hermanni: Agreement with manual count. Res Vet Sci. 2024;169:105164. doi: 10.1016/j.rvsc.2024.105164 38324973

[11] Im O, Kim S-R, Na K-J, Min K-D, Jeong D-H. Reference intervals for hematology and biochemistry in juvenile Eastern spot-billed ducks (Anas zonorhyncha) and validation of an automated hematology analyzer for avian blood analysis. PLoS One. 2025;20(10):e0334942. doi: 10.1371/journal.pone.0334942 41171862 PMC12578236

[12] Fazio F, Filiciotto F, Marafioti S, Di Stefano V, Assenza A, Placenti F, et al. Automatic analysis to assess hematological parameters in farmed gilthead sea bream (Sparus aurata) Linnaeus, 1758. Mar Freshw Behav Physiol. 2012;45(1):63–73. doi: 10.1080/10236244.2012.677559

[13] Faggio C, Piccione G, Marafioti S, Arfuso F, Trischitta F, Fortino G, et al. Monthly variations of hematological parameters of Sparus aurata and Dicentrarchus labrax reared in Mediterranean land off-shore tanks. Cah Biol Mar. 2014;55(4):437–43. doi: 10.21411/CBM.A.5F1E22E4

[14] Witeska M, Kondera E, Ługowska K, Bojarski B. Hematological methods in fish – Not only for beginners. Aquaculture. 2022;547:737498. doi: 10.1016/j.aquaculture.2021.737498

[15] Mesalles M, Uroz M, Brandts I, Serrano E, Cuenca R, Pastor J, et al. Preliminary evaluation of an automated blood cell analyzer for its use with blood samples from rainbow trout oncorhynchus mykiss. Animals (Basel). 2025;15(9):1265. doi: 10.3390/ani15091265 40362079 PMC12070899

[16] Sousa A, Pacheco A, Siqueira-Pinto G, Reis G, Fugimura M, Vaz L, et al. Comparative hematology using different anticoagulants in Colossoma macropomum anesthetized with benzocaine and eugenol by using different anticoagulants. Aquac Int. 2021;29:977–88. doi: 10.1007/s10499-021-00668-8

[17] Gonzales-Flores A, Pérez F, Quinteros K, Callejas I, Rojas J, Fernández-Méndez C. Effect of heparin and EDTA on hematology of Arapaima gigas. Aquac Int. 2022;30:263–71. doi: 10.1007/s10499-021-00796-1

[18] Jan K, Ahmed I, Sheikh Z, Fazio F. Impact of three anticoagulants and their storage time on hematological parameters of snow trout, Schizothorax labiatus, habiting in river Sindh of Indian Himalayan region. Comp Clin Pathol. 2022;31:747–55. doi: 10.1007/s00580-022-03375-9

[19] Hattingh J. Heparin and EDTA as anticoagulants for fish blood. Pflugers Arch. 1975;355:347–52. 813183 10.1007/BF00579855

[20] Sheikh ZA, Ahmed I. Comparative evaluation of two anticoagulants used for the analysis of haematological, biochemical parameters and blood cell morphology of himalayan snow trout, Schizopyge plagiostomus. Tissue Cell. 2020;67:101398. doi: 10.1016/j.tice.2020.101398 32835933

[21] Selvaraj V, Sampath K, Sekar V. Extraction and characterization of lipopolysaccharide from aeromonas hydrophila and its effects on survival and hematology of the carp, cyprinus carpio. AFS. 2004;17(2). doi: 10.33997/j.afs.2004.17.2.008

[22] Swain P, Nayak SK, Nanda PK, Dash S. Biological effects of bacterial lipopolysaccharide in fish. Fish Shellfish Immunol. 2008;25(3):191–201. doi: 10.1016/j.fsi.2008.03.013 18603445

[23] Food and Agriculture Organization of the United Nations (FAO). The State of World Fisheries and Aquaculture 2024: Blue transformation in action. Rome: FAO; 2024. doi: 10.4060/cd0683en

[24] International Council for Standardization in Haematology, Writing Group, Briggs C, Culp N, Davis B, d’Onofrio G, Zini G, et al. ICSH guidelines for the evaluation of blood cell analysers including those used for differential leucocyte and reticulocyte counting. Int J Lab Hematol. 2014;36(6):613–27. doi: 10.1111/ijlh.12201 24666725

[25] Arnold JE, Camus MS, Freeman KP, Giori L, Hooijberg EH, Jeffery U, et al. ASVCPGuidelines: Principles of quality assurance and standards for veterinary clinical pathology (version 3.0). Veterinary Clinical Pathol. 2019;48(4):542–618. doi: 10.1111/vcp.12810 31889337

[26] Parrino V, Cappello T, Costa G, Cannavà C, Sanfilippo M, Fazio F, et al. Comparative study of haematology of two teleost fish (Mugil cephalus and Carassius auratus) from different environments and feeding habits. Eur Zool J. 2018;85(1):193–9. doi: 10.1080/24750263.2018.1460694

[27] Ellis AE. The leucocytes of fish: A review. Journal of Fish Biology. 1977;11(5):453–91. doi: 10.1111/j.1095-8649.1977.tb04140.x

[28] Korcock DE, Houston AH, Gray JD. Effects of sampling conditions on selected blood variables of rainbow trout, Salmo gairdneri Richardson. Journal of Fish Biology. 1988;33(2):319–30. doi: 10.1111/j.1095-8649.1988.tb05474.x

[29] Wood BL, Andrews J, Miller S, Sabath DE. Refrigerated storage improves the stability of complete blood count results. Am J Clin Pathol. 1999;112(5):687–95. doi: 10.1093/ajcp/112.5.687 10549256

[30] Buttarello M. Quality specification in haematology: the state of the art. Clin Chim Acta. 2004;346(1):45–54. doi: 10.1016/j.cccn.2004.02.038 15234635

[31] Faggio C, Casella S, Arfuso F, Marafioti S, Piccione G, Fazio F. Effect of storage time on haematological parameters in mullet, Mugil cephalus. Cell Biochem Funct. 2013;31(5):412–6. doi: 10.1002/cbf.2915 23097308

[32] Fazio F, Ferrantelli V, Saoca C, Giangrosso G, Piccione G. Stability of haematological parameters in stored blood samples of rainbow trout Oncorhynchus mykiss (Walbaum, 1792). Vet Med. 2017;62(7):401–5. doi: 10.17221/51/2017-vetmed

[33] Laiz-Carrión R, Guerreiro PM, Fuentes J, Canario AVM, Martín Del Río MP, Mancera JM. Branchial osmoregulatory response to salinity in the gilthead sea bream, Sparus auratus. J Exp Zool A Comp Exp Biol. 2005;303(7):563–76. doi: 10.1002/jez.a.183 15945079

[34] Sysmex Veterinary Diagnostics. The influence of RBC counting technology on MCHC results. 2023. https://www.sysmex.com/en-us/training-and-knowledge/knowledge/hematology-white-paper---the-influence-of-rbc-counting

[35] Burrows A, Fletcher TC, Manning MJ. Haematology of the turbot, Psetta maxima (L.): Ultrastructural, cytochemical and morphological properties of peripheral blood leucocytes. J Appl Ichthyol. 2001;17(2):77–84. doi: 10.1046/j.1439-0426.2001.00250.x

[36] Tavares-Dias M, Barcellos JFM. Peripheral blood cells of the armored catfish Hoplosternum littorale Hancock, 1828: A morphological and cytochemical study. Braz J Morphol Sci. 2005;22(4):215–20.

[37] Vap LM, Harr KE, Arnold JE, Freeman KP, Getzy K, Lester S, et al. ASVCP quality assurance guidelines: Control of preanalytical and analytical factors for hematology for mammalian and nonmammalian species, hemostasis, and crossmatching in veterinary laboratories. Vet Clin Pathol. 2012;41(1):8–17. doi: 10.1111/j.1939-165X.2012.00413.x 22390423

[38] Walencik J, Witeska M. The effects of anticoagulants on hematological indices and blood cell morphology of common carp (Cyprinus carpio L.). Comp Biochem Physiol C Toxicol Pharmacol. 2007;146(3):331–5. doi: 10.1016/j.cbpc.2007.04.004 17509941

[39] Blaxhall PC, Daisley KW. Routine haematological methods for use with fish blood. Journal of Fish Biology. 1973;5(6):771–81. doi: 10.1111/j.1095-8649.1973.tb04510.x

[40] Witeska M, Wargocka W. Disodium EDTA used as anticoagulant causes hemolysis in common carp blood. Turk J Vet Anim Sci. 2011;35(2):99–104. doi: 10.3906/vet-0908-51

[41] Faggio C, Arfuso F, Piccione G, Zumbo A, Fazio F. Effect of three different anticoagulants and storage time on haematological parameters of Mugil cephalus (Linneaus, 1758). Turk J Fish and Aquat Sci. 2014;14(3):615–21. doi: 10.4194/1303-2712-v14_3_03

[42] Harr KE, Burnum AM, Lewis JC. Quality assurance in the hematology laboratory. Vet Clin North Am Exot Anim Pract. 2005;8(3):635–68.

[43] Pádua SB, Pilarski F, Sakabe R, Dias RA, Netto JC, Ishikawa CM. Heparin and K3EDTA as anticoagulants for tambaqui (Colossoma macropomum Cuvier, 1816). Acta Amazon. 2012;42(2):293–8. doi: 10.1590/S0044-59672012000200017

[44] Vallejos-Vidal E, Santillán-Araneda MJ, Goldstein M, Solarte-Murillo LV, Maisey K, Reyes-Cerpa S, et al. Comparison of anticoagulant vacutainer blood collection tubes on rainbow trout (Oncorhynchus mykiss) leukocyte viability during long-term storage. Fish Shellfish Immunol. 2025;161:110291. doi: 10.1016/j.fsi.2025.110291 40120780

[45] Berczi I, Bertók L, Bereznai T. Comparative studies on the toxicity of *Escherichia coli* lipopolysaccharide endotoxin in various animal species. Can J Microbiol. 1966;12(5):1070–1. doi: 10.1139/m66-143 5339644

